# A Fusion of Molecular Imprinting Technology and Siloxane Chemistry: A Way to Advanced Hybrid Nanomaterials

**DOI:** 10.3390/nano13020248

**Published:** 2023-01-06

**Authors:** Marcin Woźnica, Monika Sobiech, Piotr Luliński

**Affiliations:** Department of Organic Chemistry, Faculty of Pharmacy, Medical University of Warsaw, Banacha 1, 02-097 Warsaw, Poland

**Keywords:** molecular imprinting technology, nanomaterials, siloxanes, catalysis, detection, separation

## Abstract

Molecular imprinting technology is a well-known strategy to synthesize materials with a predetermined specificity. For fifty years, the “classical” approach assumed the creation of “memory sites” in the organic polymer matrix by a template molecule that interacts with the functional monomer prior to the polymerization and template removal. However, the phenomenon of a material’s “memory” provided by the “footprint” of the chemical entity was first observed on silica-based materials nearly a century ago. Through the years, molecular imprinting technology has attracted the attention of many scientists. Different forms of molecularly imprinted materials, even on the nanoscale, were elaborated, predominantly using organic polymers to induce the “memory”. This field has expanded quickly in recent years, providing versatile tools for the separation or detection of numerous chemical compounds or even macromolecules. In this review, we would like to emphasize the role of the molecular imprinting process in the formation of highly specific siloxane-based nanomaterials. The distinct chemistry of siloxanes provides an opportunity for the facile functionalization of the surfaces of nanomaterials, enabling us to introduce additional properties and providing a way for vast applications such as detectors or separators. It also allows for catalyzing chemical reactions providing microreactors to facilitate organic synthesis. Finally, it determines the properties of siloxanes such as biocompatibility, which opens the way to applications in drug delivery and nanomedicine. Thus, a brief outlook on the chemistry of siloxanes prior to the discussion of the current state of the art of siloxane-based imprinted nanomaterials will be provided. Those aspects will be presented in the context of practical applications in various areas of chemistry and medicine. Finally, a brief outlook of future perspectives for the field will be pointed out.

## 1. Introduction

Molecular imprinting technology is a well-known strategy that allows the synthesizing of materials with a predetermined specificity [1,2,3,4,5,6,7]. The “classical” approach, described by Wulff and Sarhan [8,9,10] in the early 1970s, assumed the creation of “memory sites” in the organic polymer matrix by a template molecule that covalently interacts with the functional monomer prior to polymerization and chemical cleavage for template residue removal. This approach, called “reversible covalent imprinting”, aims to synthesize materials that allow imitating receptors, and, as-prepared, such polymers serve as the enzyme analogs [11,12,13,14]. Moreover, the strategies of “non-covalent” and “semi-covalent” imprinting were further developed [15,16]. The former facilitates the synthesis of imprinted materials because of the employment of various types of weaker attractions between the template molecule and functional monomer during the synthesis as well as non-covalent interactions are involved in the physical adsorption of the chemical entity on the polymer. The latter assumes the incorporation of the covalently bonded structure of template monomer into the polymer matrix and, after a chemical reaction and removal of the template residue, the formation of the “sacrificial spacer”, in which the physical adsorption process occurs between the chemical compound and the polymer.

However, the phenomenon of a material’s “memory” provided by the “footprint” of the chemical compound was first observed by Polyakov [17] on silica-based materials in the early 1930s. It was noted that the presence of a “pattern molecule” during the formation of silica gels resulted in materials characterized by higher affinity to those molecules than ordinary gels. The studies performed by Pauling [18] and Dickney [19,20] aimed to explain the phenomenon and proposed possible mechanisms. Simultaneously, the further studies of Polyakov and co-workers [21,22,23] and Waksmundzki and co-workers [24,25,26,27,28,29,30,31,32], among others, contributed significantly to the development of the knowledge of silica-templated materials. Here, we would like to briefly discuss the latter works due to the significance of the findings presented therein and their low internationalization because of the poor availability of papers written mostly in native languages. In one of the papers, Waksmundzki and co-workers [24] synthesized so-called activated silica gels in the presence of various alkaloids, viz. noscapine, berberine, and cinchonine, prior to the evaluation of the adsorption capacities towards noscapine, berberine, cinchonine, papaverine, and quinine. Interestingly, it was found that not all of the “activated” gels showed specificity towards the alkaloid that was used during the synthesis. In the case of berberine, the highest adsorption was noted for the gel “activated” by cinchonine but not berberine, despite the structural differences between them. It was also found that quinine was adsorbed preferentially on the gel “activated” by cinchonine. That fact was explained by the close structural similarity of both compounds. However, it was also noted that papaverine was favorably adsorbed on the gel “activated” by noscapine, despite structural differences. It was stated that the basicity of the analyzed compounds should also be taken into account while the mechanism of adsorption was explained. In the following work dedicated to the preparation of “activated” gels towards basic compounds, viz. amines, Waksmundzki and co-workers [25] analyzed the impact of solvent on adsorption capabilities. Two different gels “activated” by 1-butylamine or cyclohexylamine were prepared together with “non-activated” control ones, and the adsorption capacities were estimated, as follows: for 1-butylamine, adsorption from water, 2 mmol g^−1^, and from cyclohexane, 4 mmol g^−1^; for cyclohexylamine, adsorption from water, 2.5 mmol g^−1^, from benzene, 3 mmol g^−1^, and from 1-propanol, 1 mmol g^−1^. Lower values of adsorption on control gels were noted for all examples, except adsorption of cyclohexylamine from 1-propanol, which was higher on the control gel than on the “activated” one. It was concluded that surface modification of gel affected specificity but the magnitude of adsorption depended on the solvent used in the adsorption process. In another work, Waksmundzki and co-workers [26] analyzed the adsorption properties of “activated” gels towards weak acids, viz. phenols, such as benzene-1,2-diol, naphthalene-2-ol, thymol (5-methyl-2-(propan-2-yl)phenol), and 8-hydroxychinoline. It was found that the gels “activated” by compounds of higher molecular weights (viz. naphthalene-2-ol) were characterized by higher specificity than gels “activated” by compounds of lower molecular weights (viz. benzene-1,2-diol). Moreover, it was found that gels “activated” by naphthalene-2-ol possessed the capability to resolute isomers of naphthalene-1-ol and naphthalene-2-ol. Interestingly, in further papers, Waksmundzki and co-workers [27,28] prepared a set of gels “activated” by pyridine, quinoline, or acridine and measured their physicochemical properties. It was found that the molecular volume of investigated compounds did not significantly affect the specific surface areas of the gels, although the increase in the pore diameter was observed as a function of the increase of the molecular volume. The multilayered adsorption was predominant in the case of pyridine adsorption from a decalin solution on an “activated” gel. Moreover, the heat of wetting and the heat of adsorption were lower for all tested “activated” gels than on the control one.

It has to be pointed out that excellent and complete outlooks of the history of molecular imprinting of silica materials were presented by Cameron and co-workers [33] as well as by Gutierrez-Climente and co-workers [34].

Throughout the years, molecular imprinting technology has attracted the attention of many scientists. Different forms of molecularly imprinted materials, even on the nanoscale, were elaborated, predominantly using organic polymers to induce the “memory”. This field has expanded quickly in recent years, providing versatile tools for the separation or detection of numerous chemical compounds or even macromolecules and cell domains, opening a wide range of applications in different branches of analytical chemistry, pharmaceutical analysis, forensic science, food control, environmental safety, and quite recently in drug delivery or theranostics. The application capabilities of molecularly imprinted materials in various fields were discussed comprehensively in numerous reviews that were published in the last few years [35,36,37,38,39,40,41,42,43,44,45,46,47,48,49,50,51,52,53,54,55,56,57,58,59,60,61,62,63,64,65,66,67,68].

**Figure 1:**
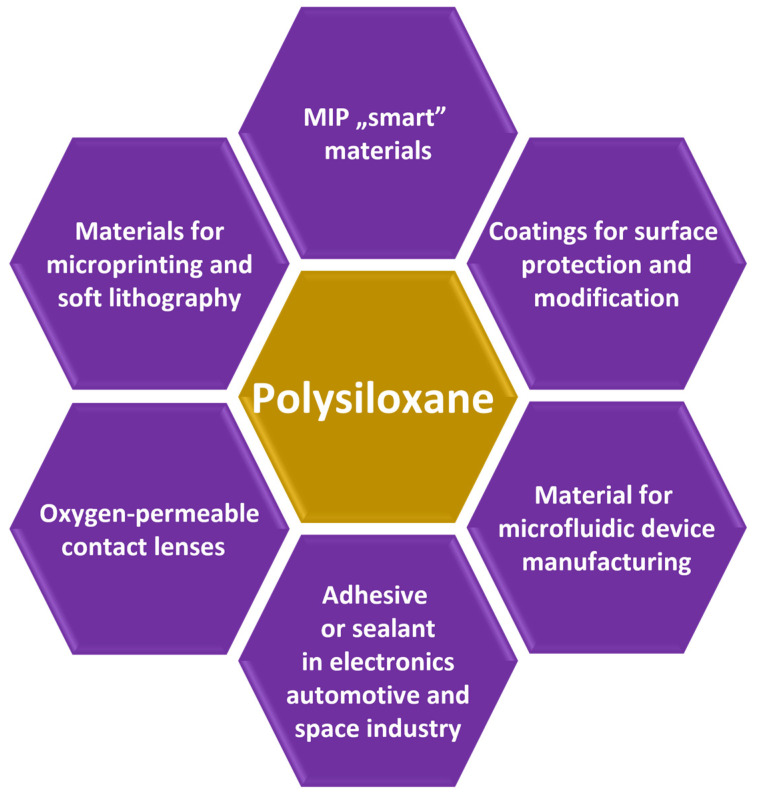
Application areas of polysiloxanes.

The utilization of the molecularly imprinted technology to organosilicones, such as siloxanes (and polysiloxanes), could significantly enhance the application capabilities of the latter materials. It should be pointed out that siloxanes have found countless and diverse applications, not only in biomedicine and pharmaceutical fields [69] but also in personal care products and cosmetics due to the non-toxicity and biocompatibility of siloxane compounds [70], as well as other areas, such as electronic manufacturing, automotive industry, textile products, construction materials, medical equipment, and food processing (Figure 1) [69,71,72,73,74,75,76,77,78,79]. It has to be underlined that such broad application capabilities of siloxanes are possible because of their extraordinary properties such as flexibility and rubber-like elasticity, fracture toughness, mechanical and chemical durability, thermal stability, compressive strength, optical transparency, and amphiphilicity, among others [80].

In this review, we would like to emphasize the role of the molecular imprinting process in the formation of highly specific siloxane-based nanomaterials. The distinct chemistry of siloxanes provides the opportunity for the functionalization of the surfaces of nanomaterials, enabling us to introduce additional properties to them, but also allowing us to catalyze chemical reactions, providing microreactors to facilitate organic synthesis. Moreover, the biocompatibility of siloxanes opens the way to applications in drug delivery and nanomedicine. Thus, a brief outlook of the chemistry of siloxanes prior to a critical discussion of the current state of the art of siloxane-based imprinted nanomaterials supported by exemplary studies and the utilization of molecularly imprinted siloxane-based materials in various areas of chemistry and medicine will be provided. Finally, outlooks and future perspectives will be pointed out.

## 2. Brief Outlook of Chemistry of Siloxanes

Siloxanes, whose name originated from the abbreviation of components of the compound, viz. silicon, oxygen, and alkanes, possess a general formula of H_3_Si(OSiH_2_)*_n_*SiH_3_, where two silicon atoms are separated by one atom of oxygen [81]. Commonly, the term “siloxanes” refers also to organosiloxanes, a group of organosilicon compounds or siloxane polymers (polysiloxanes), being alkyl or aryl derivatives of siloxanes with substituted hydrogen(s). Those compounds are also named “silicones” but nomenclature rules are ambiguous.

Taking into account the polymeric structure of siloxanes and comparing them to organic polymers, it was noted that the electron-donating capacity of the oxygen atom in the Si-O-Si system was significantly lower than in the C-O-C system instead of the fact that both elements are closely located in the periodic system [82,83]. Here, it was found that the electron pairs around the oxygen between two silicon atoms were spatially diffused with a decreased capability for electron donation. It means lower basicity of the oxygen atom in the Si-O-Si system when compared to the C-O-C system [84,85]. Moreover, the significant difference in the electronegativity between silicon-oxygen and carbon-oxygen (1.7–3.5 to 2.5–3.5, respectively, on the Pauling scale) is responsible for the high polarization of the Si-O bond [86]. The shift of electrons from oxygen to silicon atom induces the negative vicinal hyperconjugation effect which stabilizes the structure but affects the length of bonds and the angle between atoms [87,88,89]. In consequence, the Si-O bond is considered to be partially ionic in its character. The low barrier of Si-O bond rotation provides dynamic flexibility to the siloxane chain. Furthermore, the presence of alkyl or aryl groups provides an efficient shielding of the Si-O-Si backbone, resulting in weak intermolecular interactions between the polymer chains. Those factors are responsible for irregular cross-sections and the creation of free volume between the polymer constituents, which explains the low glass transition temperatures of siloxanes [90]. This fact is very important considering the construction of molecularly imprinted siloxanes since it could affect the spatial integrity of the cavity. It should also be emphasized that the various alkyl or aryl substituents in the unsymmetrical siloxane chains, which are located randomly, provide different tacticities to the polymer. Moreover, the structure of the substituent can affect the properties of siloxane’s surface, tuning its hydrophilic character. It could be used to facilitate synthesis in an aqueous environment which still is advantageous for molecularly imprinted organic polymers. On the other hand, the presence of trifluoromethyl groups increases hydrophobicity, providing the water-resistant characteristic of the resulting material [91]. The surface properties of molecularly imprinted siloxanes are very important because of solvent transfer and sorption kinetics. 

The so-called hydrolytic polycondensation is a reaction that forms polysiloxanes. This multistep reaction consists of hydrolysis and condensation processes which could undergo simultaneously [92]. The process is complex and highly dependent on the conditions that are applied. For instance, it was found that the polycondensation carried out in the presence of strong bases was greatly dominated by disproportionation processes, resulting in the random exchange of terminal siloxane units of oligomers and polymers [93]. In the case of acid-catalyzed polycondensation reaction, the protonation and the existence of hydrogen bonds with water molecules that stabilize the transition state adducts are crucial, affecting the formation of polymers or the formation of low molecular weight by-products [94].

The condensation reaction undergoes via the mechanism of nucleophilic substitution of S_N_2 type but is specific to silicon atoms, mostly due to lower repulsion effects of substituents around bigger silicon atoms (when compared to carbon) and electronic factors. Those cause changes in the S_N_2 reaction barriers characterized by different transition states (single- and triple-well potential electronic surface) to a double-well potential electronic surface with a transition state typical for S_N_2 reaction at carbon atom [95].

Hydrolytic condensation in most polymerization systems leads to amorphous polysiloxanes [96]. As Stöber [97] discovered, to obtain monodisperse siloxane microspheres, material synthesis has to be carried out in specific substrate concentration ranges and proper conditions. Modifications of the Stöber method open the way to step-by-step growth of core–shell composite multilayer materials with the ability to modify the core and/or shell of the material [98,99]. The introduction of reverse microemulsion sol–gel polymerization enables fine control of siloxane beads size in the nanometer range [100], layer composition [101], porosity [102], and morphology of grafted siloxane structures [103].

Various methods are used in the analysis and characterization of the chemical structure, microstructure, and morphology, as well as the physical properties of the siloxanes and their composites. The identification of the chemical structure of silica materials is mainly performed by the Fourier transform infrared spectroscopy and solid-state ^29^Si nuclear magnetic resonance. During Fourier transform infrared spectroscopy analysis the major peak in the range of 1000–1200 cm^−1^ that represents the asymmetric stretching vibrations of Si-O-Si bonds of silica can be observed. Besides the information on the structure of siloxanes and the degree of Si-OH condensation reaction, solid-state ^29^Si nuclear magnetic resonance can be useful during the characterization of the structures grafted at the silica surface. The microscopic structure of silica polymers and composites is often studied by X-ray techniques, such as wide-angle X-ray diffraction, wide-angle X-ray scattering, and small-angle X-ray scattering. To assess the surface compositions of the siloxanes, X-ray photoelectron spectroscopy is used [104]. Other techniques, such as differential scanning calorimetry, neutron scattering, small-angle neutron scattering, ^1^H nuclear magnetic resonance, positron annihilation lifetime spectroscopy, ^129^Xe nuclear magnetic resonance, transmission electron microscopy, scanning electron microscopy, atomic force microscopy, etc., applied for characterization of siloxane materials are described in some excellent reviews [104,105].

Based on the above-described reaction, it is possible that silane or siloxane monomers, possessing various functionalities, are able to form linear, cyclic, and polymeric structures. The synthetic approaches to siloxane polymers were comprehensively discussed in recent reviews which also disclosed a potential to synthesize more complex cage-like structures [106,107,108]. One of the interesting examples of such siloxane compounds is spherosilicates [109]. Those compounds are considered to be versatile building blocks for the fabrication of various classes of covalently linked hybrid porous polymers characterized by tunable structures and diverse properties [110]. Moreover, the capability of spherosilicates for the creation of silicon-based crown ethers was also recently described [111]. It was found that the compounds, possessing a single di-silane unit, could form stable complexes with s-block metal salts, utilizing strong tetrel bonds, with comparable coordination ability to organic crown ethers [112,113]. Those compounds can be considered extremely interesting for ion-imprinted materials, providing an alternative to ion-imprinted organic polymers [114]. 

## 3. Siloxanes as Support for Molecularly Imprinted Polymers

**Figure 2:**
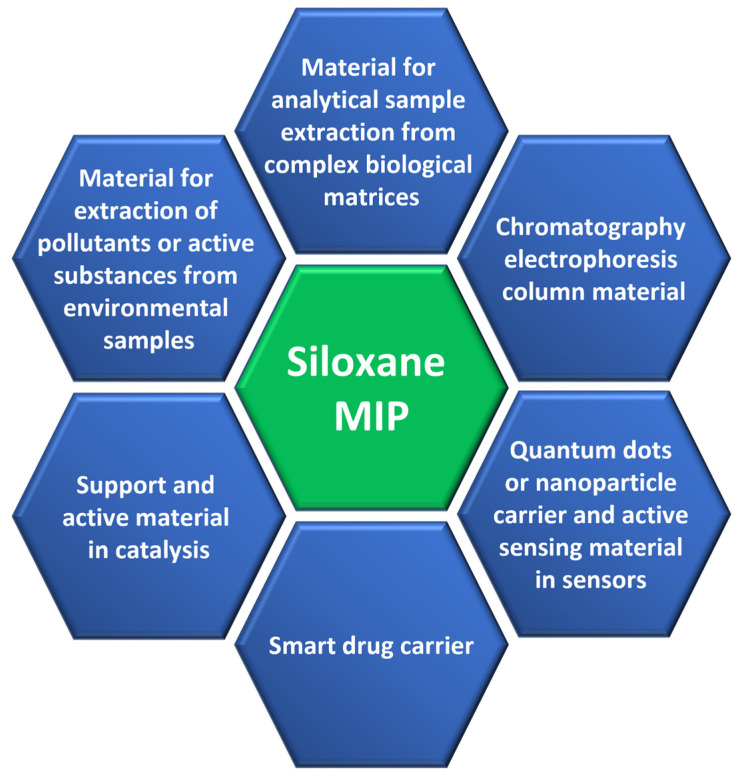
Application areas of siloxane molecularly imprinted polymers.

It has to be mentioned that the unique chemistry of siloxanes provides this class of compounds an opportunity to serve as linkers between both organic and inorganic materials [72]. Thus, we would like to point out below the potential of siloxanes as a coupling layer materials that could be functionalized on the external interface, facilitating conjugation to molecularly imprinted polymers but also a coupling layer that could be conjugated to other materials forming composites. The extent of the utilization of molecularly imprinted composites was recently summarized in a few superior reviews [45,115,116,117,118,119]. The application fields of molecularly imprinted materials where siloxanes are present as the support or imprinted matrix are shown in Figure 2. Here, we would like to focus mostly on the chemical aspects of siloxane interface functionalization, providing a platform for the development of molecularly imprinted composites.

It has to be underlined that the functionalization of the external interface of siloxanes, which is an extremely important advantage when molecularly imprinted composites are considered, is mostly realized by the presence of siloxane-derived compounds that possess unsaturated bonds, such as 3-(trimethoxysilyl)propyl methacrylate [120,121,122] or vinyltrimethoxysilane [123] after the hydrolysis/condensation processes. In one of the advanced approaches, Song and co-workers [124] utilized 3-(trimethoxysilyl)propyl methacrylate to covalently bond octavinyl-modified polyhedral oligomeric silsesquioxanes. This strategy allowed obtaining siloxane-based star-shaped support for further conjugation with organic molecularly imprinted shell with improved site accessibility and capacity for enantiomeric separation of S-amlodipine. Such dendrimeric structures are advantageous when compared to linear counterparts because they provide faster mass transfer, better accessibility to specific sites, and, in consequence, enhance selectivity, the properties highly demanded in the fabrication of molecularly imprinted materials.

Nevertheless, very interesting approaches utilizing the potential of the chemistry of siloxanes were also recently presented. Li and co-workers [125] proposed a functionalization of silica-based particles by 3-(aminopropyl)triethoxysilane to provide an amine group on the external interface. Next, the reaction with 4-cyano-4-(phenylcarbonothioylthio)pentanoic acid, an agent that induces the reversible addition–fragmentation chain transfer process, on the amine group was carried out, yielding the formation of sulfide bonds with siloxane residue. The process called “grafting” allowed co-polymerizing methacrylic acid, 2-hydroxyethyl methacrylate, and N,N-methylenebisacrylamide to obtain the molecularly imprinted material for recognition of lysozyme. The strategy could be easily extended for the implementation of other functional monomers which could be used in the future to fabricate more specific imprinted materials [126]. Nevertheless, attention should be paid to the constitutive release of hydrogen sulfide from the dithiobenzoate group attached to siloxane in certain biological systems, limiting practical application in the biomedical field [127]. Xu and co-workers [128] used 3-(aminopropyl)triethoxysilane to functionalize the surface of tetraethoxysilane followed by the reaction with maleic anhydride to obtain 3-(triethoxylsilylpropylcarbamoyl)crotonic acid, introducing unsaturated bonds capable of polymerizing in the presence of methacrylic acid and ethylene glycol dimethacrylate. This strategy allowed the introduction of additional amide and carboxylic groups to enhance the binding capacity of imprinted material during the adsorption of proteins [129]. Similarly, Cheng and co-workers [130] used 3-(aminopropyl)triethoxysilane to functionalize the surface of tetraethoxysilane followed by the reaction with methacryloyl chloride to provide an unsaturated bond prior to the polymerization reaction of organic monomers to form molecularly imprinted layer specific to quercetin. Zhao and co-workers [131] utilized 3-(aminopropyl)triethoxysilane to covalently attach 4,4′-azobis(4-cyanopentanoic acid), an initiator of the radical polymerization, by the formation of amide linkage either directly or after subsequent reaction of 4,4′-azobis(4-cyanopentanoic acid) with thionyl chloride. This approach allowed for polymerizing a thin organic layer of imprinted polymer, facilitating the sorption process. Nevertheless, it was found that the yield of grafting was very low due to the moderate availability of amine groups. This approach was previously extended by Sulitzky and co-workers [132] for the application of 3-(glycidoxypropyl)trimethoxysilane to a functional silica-based surface followed by the cleavage of the epoxy ring. Recently, new siloxane-derived initiators for surface tethering were synthesized, opening the way for applications in the fabrication of imprinted materials [133]. 

**Figure 3:**
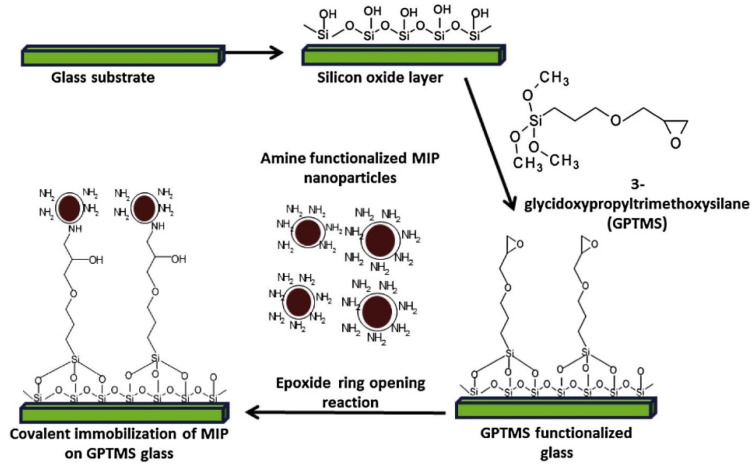
Process of immobilization of core–shell MIP nanoparticles. Reprint from Elsevier [134].

A very interesting approach was proposed by Kamra and co-workers [134], who proposed covalent immobilization of propranolol-imprinted polymer nanoparticles using an epoxy-derived siloxane (Figure 3). The nucleophilic substitution after the epoxide ring opening was enabled due to the presence of allylamine residues on the surface of molecularly imprinted particles. It was proved that covalent bonds between functionalized siloxane and imprinted nanoparticles significantly improved the stability and analytical performance of the sensor. It was stated that the method was applicable to various amine functionalized materials for lab-on-chip and other sensing applications and could be easily extended to other imprinted materials without deterioration of their specificity.

Another aspect of the supporting role of siloxanes is their application to solid-phase synthesis of molecularly imprinted organic polymers. One of the excellent papers that used the covalent strategy to attach the template to the siloxane-based monomer in solid-phase synthesis was recently published by Piletsky and co-workers [135]. In this work, (3-iodopropyl)trimethoxysilane monomer was employed to bond covalently a mercapto-derived peptide, acting as the template, aiming to introduce specific recognition sites to the materials towards epitopes of epidermal growth factor receptor, a cancer biomarker. Prior to the nucleophilic substitution of the mercapto group, (3-iodopropyl)trimethoxysilane was attached to the silicon oxide support by the hydrolysis/condensation process. In alternative routes, (3-aminopropyl)triethoxysilane or N-(6-aminohexyl)aminomethyltriethoxysilane were firstly attached to silicon oxide support followed by the reaction of the amine group with succinimidyl iodoacetate and the nucleophilic substitution of the mercapto group of peptides. Then, the polymerization of the organic polymer overlayer was processed, templating the epitope. The resulting molecularly imprinted organic polymers were characterized by satisfactory specificity. It was concluded that the use of (3-iodopropyl)trimethoxysilane was advantageous because of the reduction of synthetic steps and costs but also due to the elimination of disadvantages derived from the presence of an amine group, such as unintended side reactions and poorly controlled polymerization. It was also stated that alkyl halides, such as (3-iodopropyl)trimethoxysilane could be very useful reagents for synthesis with other nucleophilic groups beyond thiols.

The above-mentioned examples show the capability of selected siloxane reagents to form support materials for molecular imprinting but the full potential of functionalized siloxane and polysiloxanes, such as docylmethylsiloxane–(hydroxyl alkyleneoxypropyl)–methylsiloxane or α,ώ-divinyl–poly(dimethylsiloxane) among others, was revealed in recent reviews [136,137,138]. This opens a broad way to applications as new reagents for molecular imprinting as well.

It should be pointed out here that siloxanes could serve as support compounds that improve certain properties of molecularly imprinted polymers. Zhang and co-workers [139] applied vinyl-derived polyhedral oligomeric silsesquioxane to suppress the non-specific adsorption on the molecularly imprinted polymer that served as drug carriers for paclitaxel delivery. The effective suppression of non-specific adsorption is a very important goal considering molecularly imprinted drug carriers because it could be responsible for the burst effect. In further investigations, vinyl-derived polyhedral oligomeric silsesquioxanes were applied together with mesogenic compounds to optimize the construction of a floating molecularly imprinted drug delivery system [140,141].

Siloxane reagents play a crucial role in the conjugation of other surfaces. Among them, silicon oxide nanoparticles as well as silica-based mesoporous systems are mostly utilized, providing internal silicon-derived complex systems. The nature of silicon oxide enables the adsorption of water forming equilibrium states, possessing hydroxy groups and silicic acid [142]. Thus, the aqueous environment plays an important role prior to the covalent bonding of functionalized siloxanes. In the aprotic solvent, the weakened hydrogen bonds could dominate in the interactions with the surface [143].

The capability of silicon oxide to form regular and homogeneous spheres was utilized to fabricate silica/siloxane-based supports for molecularly imprinted organic polymer stationary phases for chromatography and sorbents for solid-phase extraction. Here, the literature survey disclosed an extensive number of examples, which were comprehensively discussed in a few excellent reviews [50,144,145,146,147,148,149,150]. Thus, these aspects will be omitted here but we would like to focus a little attention on silica-based mesoporous materials.

Silica-based mesoporous materials are extensively investigated as an area for functionalization by siloxane to fabricate molecularly imprinted materials. Numerous classes of silica-based mesoporous materials were synthesized, providing a large surface area, large pore size, an abundance of surface functional groups, and stable physicochemical properties. Those properties made them very promising tools for molecular imprinting and the potential of silica-based mesoporous molecularly imprinted materials was revealed recently in a few excellent reviews [151,152,153]. As a recent example, An and co-workers [154] used mesoporous silica material, viz. SBA-15, to improve the adsorption behavior of the resulting molecularly imprinted material. The functionalization of mesoporous support was provided by 3-(trimethoxysilyl)propyl methacrylate before the covalent attachment of a prepolymerization mixture of methacrylic acid, zinc acrylate, and ethylene glycol dimethacrylate in the presence of glutarylated pentapeptide. In order to improve the homogeneity of the SBA-15 functionalized material in the prepolymerization mixture, the mixture of choline chloride and ethylene glycol acting as the deep eutectic solvent was employed. It has to be underlined that, in the preparation step, siloxanes serve as the coating layer of silica-based mesoporous material to which the molecularly imprinted shell is attached via covalent bonds. This process, although it provides specificity to the material, resulted in a significant decrease in the specific surface area of the initial mesoporous material. 

It has to be emphasized that the above-discussed processes of siloxane conjugations are very often extended to interfaces of other initial materials in which many of them are on the nanoscale, such as spherical dots, planar sheets, and cubic tubes. Carbon-based conjugates of siloxanes in the form of carbon dots, graphene sheets, and multi-walled carbon nanotubes could be examples because of the unique properties of carbon materials. For instance, carbon dots, graphene quantum dots, and carbon quantum dots were used as initial materials for conjugation with tetraethoxysilane and (3-aminopropyl)triethoxysilane to obtain specific photoluminescence materials [155,156,157]. In another paper, Li and co-workers [158] elaborated on a siloxane composite of reduced graphene oxide and tetraethoxysilane/3-(aminopropyl)triethoxysilane with silver nanoparticles as support for modification with a polydopamine layer. Then, the coating with an external molecularly imprinted shell composed of methacrylic acid and acrylamide was proposed. The composite was developed for the surface-enhanced Raman scattering of λ-cyhalothrin detection, and the extraordinary electronic properties of reduced graphene oxide such as background noise elimination enhanced the analytical performance of the resulting detector. Here, the crucial step involved the effective attachment of graphene form to siloxanes, which, in most cases, required a reaction between the carboxylic group of functionalized graphene and the amine group of 3-(aminopropyl)triethoxysilane. The main disadvantage of this step was a proper selection of reagents due to the facile aggregation of graphene forms, which limited the yields of the reaction [159]. It has to be mentioned that other forms of carbon or carbon derivatives were also utilized during the formation of siloxane support for molecular imprinting, for example, graphitic carbon nitride and carbon nanotubes [160,161].

Apart from silicon oxide and carbon-based forms, siloxanes are frequently used to conjugate interfaces of other materials. It opens a way to introduce additional properties to the resulting molecularly imprinted composites. The composition diversity of those materials is huge, spanning from homogeneous gold particles to more complex metal oxides and to, for instance, ternary quantum dots. Taking into account the diversity of materials, we have limited here a discussion to an exemplary composite of magnetite siloxane because of its predominant role in the fabrication of molecularly imprinted composites. Here, we would like to focus on the problems related to the homogeneity of nanomaterials, which could affect the conjugation of siloxanes.

Magnetic nanoparticles are one of the most frequently utilized initial nanomaterials to form, as a final result, a molecularly imprinted composite [162]. Moreover, conjugation to the siloxane layer is a preferable approach before final modification by the molecularly imprinted polymer. However, factors such as the molar ratio of iron salts, the pH of the solution, temperature, stirring velocity, and the presence of stabilizers affect the homogeneity and surface properties of magnetic nanoparticles [163]. These parameters alter their size and, in consequence, affect the capability of mutual aggregation in order to reduce the surface energy and affect the surface electrostatic charge. This leads to the precipitation of magnetite with co-precipitates such as maghemite, hematite, or even goethite, decreasing the magnetic saturation values of nanomaterials and tuning interface properties [164,165,166]. Taking into account a magnetic saturation value of magnetic molecularly imprinted composites as a comparable parameter, it was found that the magnetic saturation of nanoparticles formed when only the same system was applied varied significantly from 37.5 emu g^−1^ [167] to 82.2 emu g^−1^ [130]. The surface disorders, spin canting, and random orientation of spin moments on the surface could explain the variations [168]. Nevertheless, the results indicate substantial differences in the structure and composition of magnetic nanomaterials which could significantly affect the further process of functionalization with siloxane. Moreover, it should be emphasized that the simultaneous formation of neat (without magnetic nanoparticles) siloxane particles is an inevitable process, enforcing additional further steps for removing them. The phenomenon is explained by the increase of the ionic strength of the solution during the tetraethoxysilane hydrolysis, causing the increase of electrostatic interactions between the surfaces of magnetic nanoparticles and aggregation. As a result, the number of magnetic cores as nucleation sites decreases, promoting the silica nucleation rate [169]. It should be also underlined that this process could lead to the physical occlusion of magnetic cores by growing siloxane polymer, causing facile degradation of such structures during the analysis of molecularly imprinted composites and loss of magnetic properties. In our recent studies, we have investigated the effect of the composition of magnetic-siloxane nanoparticles on the magnetic property, stability, and selectivity of molecularly imprinted composites, taking into account various ratios of magnetic-siloxane nanoparticles to organic prepolymerization mixture [170]. It was found the magnetization value was saturated for samples with a content of more than 15% of magnetic-siloxane nanoparticles. It was also concluded that the higher the amount of siloxane functionalized core, the thinner the molecularly imprinted shell layer. The thickness of the imprinted layer affected the magnetic properties of the composite but did not affect the sorption process which was limited to the surface of the composite.

Lastly, we would like to briefly address gold–siloxane conjugates. Gold nanoparticles or thin layers are frequently used in the fabrication of molecularly imprinted composites for electrochemical or optical detection purposes. As a noble metal, gold does not form oxides at its surface, thus direct hydrolysis/condensation process to functionalize the gold interface is impossible. Mostly, primers are required to promote the siloxane layer coating. On the other hand, gold nanoparticles have to be stabilized by, for example, citrate ions, which could be substituted by siloxane primers, such as 3-(aminopropyl)triethoxysilane or 3-(mercaptopropyl)triethoxysilane, utilizing various weak attractions between nitrogen or sulfur atoms with the gold surface [171]. For example, Leite and co-workers [172] applied 3-(mercaptopropyl)triethoxysilane as a primer before the hydrolysis/condensation of 3-(aminopropyl)triethoxysilane, phenyltriethoxysilane, and tetraethoxysilane to form siloxane-based molecularly imprinted electrode surface for detection of caffeic acid.

It should be noted that approaches dedicated to siloxanes that serve only as functional monomers in the molecular imprinting process of the siloxane layer will be discussed further below.

The proper conjugation of the internal interface of siloxanes as well as the effective functionalization of the external interface of siloxane ensure the homogeneity, stability, and durability of resulting materials. 

## 4. Siloxane-Based Molecular Imprinting for Catalysis

The geometry of surface regions templated by the molecule together with the complementary distribution of the electrostatic potentials enables molecularly imprinted polymers to serve as catalysts. The involvement of metal complexes of templates allows the preparation of coordinatively unsaturated catalytic active sites. A lot of effort was undertaken to design molecularly imprinted catalysts to imitate artificial enzymatic catalyst systems with catalytic properties close to those of enzymes. Mostly, organic molecularly imprinted polymers were explored for such a purpose, but the potential of siloxane-based materials to form specific catalytic sites was also revealed and recently reviewed [173,174,175]. Most prominent examples that showed the utility of siloxane-based imprinted materials for catalysis were described by Murastugu and co-workers [176,177,178,179,180,181]. In one recent paper, Muratsugu and co-workers [176] described the fabrication of a molecularly imprinted polysiloxane material that possessed specific regions, “cavities” for cross-coupling Suzuki reactions. A stereochemically dependent Suzuki reaction between aryl halides and arylboronic acids is very important in organic chemistry because it enables the formation of C-C bonds, mostly in the synthesis of unsymmetrical biaryl compounds. In order to catalyze the reactions, the palladium adduct of 5-(2-propen-1-ol)-[1,1′-biphenyl]-2,2′-diol and ethane-1,2-diylbis[bis [4-(1-methylethenyl)phenyl]-phosphine] was obtained and coupled to siloxane surface functionalized by vinylphenyltrimethoxysilane. Once the complex was coupled to support, the overlayer was formed around by the reaction of tetramethoxysilane in the presence of triethoxy-3-(2-imidazolin-1-yl)propylsilane, which promoted the hydrolysis/polymerization reaction and produced rigid siloxane matrix. It was found that the thickness of the siloxane overlayer played a crucial role in the catalysis of the Suzuki coupling and decrease the ratio of conversion of reagents with higher molecular volume. A quite similar strategy was utilized to examine the spatial effects and catalytic activity of ruthenium–porphyrin-(triethoxysilyl)propylcarbamate complex in the epoxidation reaction of cholesterol derivatives [177] and ruthenium–N-4-styrenesulfonyl-1,2-diphenylethylenediamine complex in the hydrogenation of 2-fluorobenzophenone or the regioselective epoxidation of (R)-(+)-limonene, among others [178,179,180,181].

The role of 3-(aminopropyl)triethoxysilane to catalyze the condensation reaction, instead of the conventional basic catalysts, was explored by Wang and co-workers [182]. It was found that the absence of additional catalysts during the molecular imprinting of siloxanes eliminated the detrimental effect on the imprinting performance. The prepolymerization mixture included 3-(aminopropyl)triethoxysilane, acting as the functional monomer and catalyst; tetraethoxysilane, the cross-linker; and 1-naphthyl phosphate, a template. The resulting material was characterized by acceptable specificity but the yields of the condensation process were moderate. In another exemplary paper, Huo and co-workers [183] described molecularly imprinted hybrid mesoporous material with catalytic activity towards the Knoevenagel reaction, an important synthetic route to form C-C bonds, involving aldehydes as reagents. In the material’s fabrication process, (3-isocyanatopropyl)triethoxysilane reacted with benzene-1,4-diol or 2,2-di-(4-hydroxyphenyl)propane to form siloxane-derived amides that were further cross-linked by tetraethoxysilane. After that, the hydrolysis of amide linkage was carried out to leave the amine residues in the siloxane “cavity”. The catalytic performance was estimated by the Knoevenagel reaction of 4-methoxybenzaldehyde and naphtalene-1-carboaldehyde, and the capability to catalyze the reaction was proved.

Finally, the first attempt at biomimetic siloxane bond formation catalyzed by molecularly imprinted organic polymers was described by Abbate and co-workers [184]. In that study, a model siloxane compound of trimethylethoxysilane was used for examination of the condensation reaction under mild conditions with diphenylsilanediol acting as the “pseudo-transition state analog”. It was assumed that a pentacoordinate silicon intermediate was generated during the nucleophilic attack of the oxygen of a molecule of silanol on the silicon atom of the second silanol molecule. The two hydroxyl groups of the imprinted molecule could interact through hydrogen bonds to the carboxy groups of methacrylic acid residues, while the phenyl rings could participate in non-polar interactions with the apolar region of the polymer matrix.

## 5. Molecularly Imprinted Siloxane Layers

In this section, we will discuss the approaches to form molecularly imprinted siloxane materials utilizing covalent and non-covalent strategies, and we will present the applications of those materials for the separation, detection, and potential drug delivery purposes.

The covalent attachment of the template to siloxane-based monomers is occasionally utilized to form molecularly imprinted siloxane polymers. In a unique recent paper, Effting and co-workers [185] created a “sacrificial spacer” for the non-covalent adsorption of the target molecule cholesterol. The strategy involved the formation of a siloxane layer composed of tetraethoxysilane and cholesterol-functionalized siloxane compound that was obtained by the reaction between 3-(triethoxysilyl)propyl isocyanate and hydroxy group in position 3 of sterol system, enabling the formation of the amide bond. The removal of the sterol system was proceeded by the hydrolysis of amide linkage in dimethyl sulfoxide to avoid hydrolysis of siloxane structure, leaving amine residue in the siloxane network. It allowed for the non-covalent adsorption of cholesterol, the process which was most effective from hexane.

The non-covalent strategy is an approach mostly utilized to fabricate molecularly imprinted siloxane materials. Various siloxane-based functional monomers were investigated to form stable adducts with templates in mono-monomer systems, such as 3-(aminopropyl)trimethoxysilane [186] and dual-monomer systems such as phenyltrimethoxysilane and 3-(aminopropyl)trimethoxysilane [187] and phenyltriethoxysilane and 3-(aminopropyl)trimethoxysilane [172]. Some recent papers that describe siloxane-based imprinted materials together with the application purposes of the resulting materials are presented in Table 1.

**Figure 4:**
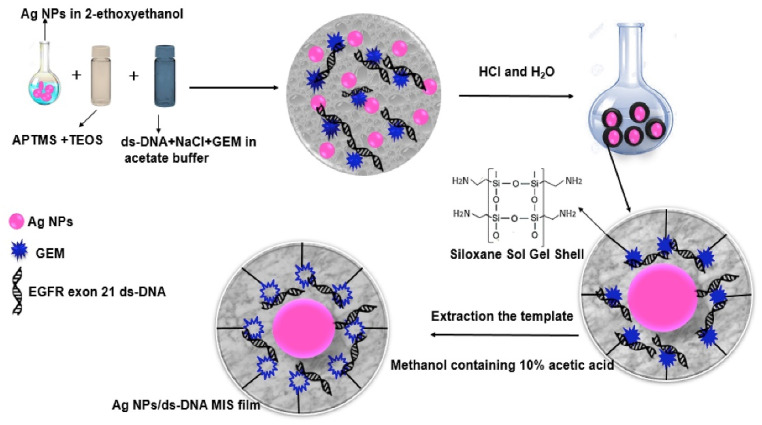
Synthetic steps of molecularly bio-imprinted siloxane biosensor on the basis of core–shell silver nanoparticles/EGFR exon 21 L858R point mutant gene/siloxane film. Reprint from Elsevier [219].

One of the interesting concepts was presented by Shoja and co-workers [219] who elaborated on the electrochemical molecularly bio-imprinted siloxane biosensors on the basis of core–shell silver nanoparticles/EGFR exon 21 L858R point mutant gene/siloxane film for ultra-sensing of gemcitabine as a lung cancer chemotherapy medication (Figure 4).

Biosensors composed of siloxane-based materials are characterized by chemical inertness, physical rigidity, thermal stability, high surface area and porosity, compatibility with an aqueous environment, facile fabrication under mild conditions, and, moreover, a lack of electrocatalytic activities and conductivities when compared to most organic polymers. Taking the above advantages into account, it was possible to elaborate on biosensors for DNA immobilization, offering interesting electrocatalytic properties for electrochemical investigations. Under optimized conditions, the biosensor provided suitable stability, wide linear range, short response time, and low detection limit, revealing the potential of siloxane-based materials for biomedical and clinical applications. These aspects were also comprehensively discussed in an excellent review by Gutierrez-Climente and co-workers [34].

Next, we would like to address the capability of molecularly imprinted siloxanes to serve as potential drug delivery vehicles. In a rare approach, Li and co-workers [211] presented an investigation that aimed to fabricate water-compatible silica sol–gel molecularly imprinted polymer as a potential delivery system for the controlled release of salicylic acid. The molecularly imprinted layer was composed of stoichiometric amounts of functional monomers, 1-(4-vinylphenyl)-3-(3,5-bis(trifluoromethyl)phenyl)urea 3-(aminopropyl)triethoxysilane, trimethoxyphenylsilane, and a tetraethoxysilane cross-linker. In vitro release studies in aqueous systems revealed the results that were modeled by Fick’s law of diffusion, showing the diffusion-controlled release mechanism as predominant.

## 6. Comparison of MIPs Based on Siloxanes and Organic Polymers

Finally, we would like to discuss the effectiveness of the imprinting process on siloxane-based materials when compared to organic molecularly imprinted polymers. Here, Lafarge and co-workers [210] explored the sorption properties of molecularly imprinted siloxane and molecularly imprinted organic polymer towards the target analyte, iprodione (3-(3,5-dichlorophenyl)-2,4-dioxo-N-(propan-2-yl)imidazolidine-1-carboxamide). For that purpose, a siloxane-based polymer composed from a tetraethoxysilane cross-linker and 3-(aminopropyl)triethoxysilane, was prepared and compared to an organic-based polymer, a copolymer of poly(allylamine-co-ethylene glycol dimethacrylate). The adsorption studies were processed from aqueous ethanol solutions. In both materials, amine residues in the polymer network could interact with the analyte during the sorption studies. It was found that the siloxane-based polymer was characterized by incomparably high specificity at the level of 135 times when compared to its organic counterpart but the overall binding capacity was halved lower than on organic-based polymer. It was hypothesized that the high homogeneity of the siloxane-based material explained the high specificity. Data from the Freundlich model revealed the following values of heterogeneity indices for siloxane-based and organic-based polymers: 0.86 and 0.47, respectively. It was also found that siloxane-based non-imprinted counterparts adsorbed practically none. In conclusion, it was stated that the siloxane-based material could specifically adsorb analyte even in an extremely polar environment in contrast to organic polymer materials. On the contrary, the organic-based polymer, when applied to solid-phase extraction as a sorbent, was characterized by significantly higher sensitivity, allowing the use of shorter extraction columns and lower volumes of samples for analysis.

## 7. Outlook and Future Prospects

The merger of molecular imprinting technology and siloxane chemistry provides a facile way to fabricate advanced hybrid nanomaterials with high specificity and selectivity that could find applications in the separation and detection of various molecules. Additionally, the production of composites, where imprinted polymers are conjugated by the silica layer to the other materials such as magnetic particles or quantum dots, makes it possible to use these materials in biomedical applications, viz. drug delivery or bioimaging. The third way of siloxane-based imprinted forms utilization is enzyme-like catalysis. Silica compounds are used according to imprinting technology in three different ways, as conjugation/functionalization layers during the formation of composite material, as cores in the creation of the core–shell particles, and for the production of the imprinted matrix. Except for supporting roles, siloxane could improve the specificity and adsorption properties of molecularly imprinted polymers as well as improve the stability and analytical performance of the obtained imprinted sensors. Nevertheless, the problems relevant to the synthetic process of silica-contained imprinted materials need to be eliminated or reduced. The heterogeneity of composite materials, low grafting level of the imprinted layer to silica shell, and lower capacities of silica-based imprinted polymers in comparison with the organic matrices are the main difficulties. The effect of solvent is also very important due to its role in the creation of macropores or hierarchical pore structures, allowing not only to enhance the adsorption capacity and increase the specificity but also to obtain other forms of siloxane-based imprinted materials such as membranes. However, the precursor-solvent interactions in sol–gel reactions have not been extensively investigated. In our opinion, the explanation of the interactions between silica precursors, co-precursors, solvents, catalysts, and polymers is very important to understand spinodal decomposition, a process that affects the strength of silica materials. Modification of the synthetic strategies with the use of new siloxane-derived initiators, new reagents, and new synthetic methods could be a solution to existing problems. Here, the following particular approaches could be identified: (a) the introduction of more advanced siloxane-based compounds for the synthesis of siloxane-based imprinted materials, for instance, silatrane derivatives; (b) the use of spherosilicates to obtain siloxane-based imprinted materials for ion separation; (c) the application of novel mesoporous materials as supports for siloxane-based imprinted materials, such as KIT-6 or MCM-48. Despite that, silica-contained imprinted materials have great potential for use in a wide range of different areas, such as analytical approaches, drug delivery systems, and catalysis.

## Figures and Tables

**Table 1:** Recent examples of siloxane-based imprinted materials, compositions, polymerization methods, target molecules, material forms, and applications.

Material Composition	Polymerization Method	Target Molecule	Material Form	Application	Ref.
T: L-Tryptophan/ D-tryptophan M: APTES C: TEOS S: ITO electrode Brij58	Acidic sol–gel polymerization on immersed ITO electrode	L-Tryptophan, D-tryptophan	Polymer film on ITO electrode	Electrochemical chiral recognition in buffer solution	[103]
T: 2,4,6-trichlorophenol M: APTES C: TEOS S: CdSe@CdS QDs Triton X-100	Reverse microemulsion polymerization	2,4,6-trichlorophenol	MIP silica microspheres-coated CdSe@CdS QDs	Fluorescent sensor for environmental water analysis	[101]
T: Bisphenol A M: BIMS C: TEOS CTAB	Basic sol–gel polymerization	Bisphenol A	Imprinted mesoporous silica	Selective removal of bisphenol A from wastewater	[102]
T: Entacapone M: APTES C: TEOS S: GO QDs	Basic sol–gel polymerization	Entacapone	Molecularly imprinted silica coated on GO QDs	Fluorescent nanosensors for pharmaceutical samples	[187]
T: Malachite Green M: APTES C: TEOS S: C_3_N_4_ QDs, CdTe QDs CTAB	Basic sol–gel polymerization	Malachite Green	Mesoporous silica MIP coated on C_3_N_4_ and CdTe QDs	Dual-emission fluorescent probe for fish farming water analysis	[188]
T: Fipronil M: APTES C: TEOS S: CDs	Basic sol–gel polymerization	Fipronil	Silica MIP coated on CDs	Fluorometric probe for fipronil in spiked eggs, milk, and tap water	[189]
T: Theobromine M: APTES C: TEOS S: Au@Ag nanorods CTAB	Basic sol–gel polymerization	Theobromine	Multishell Au@Ag@SiO_2_ nanorods coated with MIP	Electrochemical sensor for quantification of theobromine in food, biological, and environmental samples	[190]
T: Serotonin M: PTMOS C: TEOS S: Fe_3_O_4_@Au	Sol–gel polymerization	Serotonin	Graphite screen-printed electrode modified with Fe_3_O_4_@Au@SiO_2_@MIP	Selective paper-based electrochemical electrode for serotonin determination in dietary supplement capsules and urine	[191]
T: Difenoconazole M: APTES/PTMOS C: TEOS CTAB	Sol–gel polymerization	Difenoconazole	Silica MIP coating on Nichrome wire	MIP solid-phase microextraction of difenoconazole from wheat and fruits samples for gas chromatography	[192]
T: Amifostine M: APTES/CSPTMS C: TEOS S: CDs	Sol–gel polymerization	Amifostine, alcaline phosphatase	Silica MIP coated on CDs	Fluorometric sensor for amifostine in human serum	[193]
T: S-amlodipine M: MAA C: TEOS S: MOF-177	Acidic sol–gel polymerization	S-amlodipine	Metal framework core-organic/inorganic hybrid shell material	S-amlodipine solution	[194]
T: Thiabendazole M: APTES C: TEOS S: CDs	Reverse microemulsion polymerization	Thiabendazole	Silica MIP coated on CDs	Fluorometric sensor for determination of thiabendazole in juices	[195]
T: 1,10-phenanthroline- 4-carboxylic acid M: APTES C: TEOS	Acidic sol–gel polymerization	Aristolochic acid I	Silica MIP gel	Removal of aristolochic acid I from kaempfer dutchmanspipe root extract	[96]
T: Phoxim M: BUPTEOS C: TEOS S: CsPbBr_3_ Perovskite QDs	Hydrolysis	Phoxim	Perovskite QDs coated with MIP	Fluorescent sensor for Phoxim detection in potato and soil sample	[196]
T: Sulfamethoxazole M: APTES C: TEOS S: Agarose gel	Acidic sol–gel surface polymerization on silica gel cores	Sulfamethoxazole, sulfamonomethoxine, sulfadiazine	Membrane consisting of silicon MIP dispersed in an agarose matrix	Separation and preconcentration of sulfonamide antibiotics in water samples	[197]
T: TBBPA M: ICPTES/GYLMO C: TEOS S: Fe_3_O_4_ P123	Acidic sol–gel polymerization	Bisphenol A	Silica MIP gel with incorporated Fe_3_O_4_	Extraction of Bisphenol A from water samples	[198]
T: Acetylthiocholine M: APTES C: TEOS S: CDs@AEAPMS	Basic sol–gel polymerization	Acetylcholinesterase	MIP coated CDs composite	Fluorescence detection of acetylcholinesterase	[199]
T: Difenoconazole M: APTES C: TEOS S: CdTe QDs@ glutatione	Sol–gel polymerization	Difenoconazole	Silica MIP coated CdTe QDs	Fluorescent sensor for detecting and determining difenoconazole in agricultural products	[200]
T: Cefrazidime M: APTES C: TEOS S: CDs	Basic sol–gel polymerization	Ceftazidime	CDs embedded in silica MIP	Nanosensor to analyze ceftazidime in urine samples	[201]
T: Bisphenol A, 4-cumylphenol M: APTES, PTMOS C: TEOS S: Fe_3_O_4_@SiO_2_	Basic sol–gel polymerization on Fe_3_O_4_@SiO_2_ cores	Bisphenol A, 4-cumylphenol	Magnetic silica core–shell MIP	Selective detection of phenolic endocrine disrupting compounds in foodstuffs	[98]
T: Diazinon/ Monocrotophos M: APTES/PTMOS C: TEOS	Basic sol–gel polymerization	Dimethoate, fenthion sulfoxide, fenthion sulfone, methidathion, malathion, fenitrothion, diazinon, pirimiphosmethyl, fenthion, chlorpyrifosethyl, and monocrotophos	Silica MIP gel	Selective extraction of polar organophosphorus pesticides from almond oil	[202]
T: Nonylphenol M: NP-3-APTMOS C: TEOS CTAB	Basic sol–gel polymerization	Nonylphenol	Mesoporous silica MIP	Solid-phase extraction of nonylphenol in textile samples	[203]
T: 1-Naphthyl phosphate M: APTES C: TEOS	Basic sol–gel polymerization—APTES catalyzed	1-Naphtyl phosphate, naproxen, benzoic acid	Silica MIP gel	Solid-phase microextraction from water samples	[182]
T: Naproxen M: APTES, FITC- APTES C: TEOS	Basic sol–gel polymerization—APTES catalyzed	Naproxen	Silica MIP gel	Detection of naproxen in spiked tap water	[66]
T: S-naproxen M: APTES C: TEOS S: CDs	Basic sol–gel polymerization	Naproxen	CDs embedded in silica MIP	Fluorescent nanoprobe for enantioselective quantification of naproxen enantiomers in pharmaceutical samples	[155]
T: Thioridazine hydrochloride M: APTES C: TEOS S: ZnO QDs Triton X-100	Basic reverse microemulsion sol–gel polymerization	Thioridazine hydrochloride	ZnO QDs coated with silica MIP	Fluorescent optical sensor for thioridazine hydrochloride detection in plasma samples	[204]
T: Theophylline M: PTEOS C: TEOS CTAB	Sol–gel polymerization and electrochemical polypyrrole polymerization coated on GCE	Theophylline	Mesoporous silica spheres polypyrrole hybrid on electrode surface	Electrochemical quantification of theophylline in green tea, carbonated cola drink, fermented milk drink, and preserved fruit	[205]
T: Chlorogenic acid M: APTES C: TEOS S: ZnO QDs Triton X-100	Reverse microemulsion polymerization	Chlorogenic Acid	ZnO QDs coated with silica MIP	Fluorometric sensor for chlorogenic acid quantification in spiked human plasma samples	[206]
T: Omethoate M: APTES C: TMOS S: CsPbBr_3_ perovskite	Sol–gel polymerization	Omethoate	CsPbBr_3_ perovskite coated with silica MIP	Fluorescent sensor for omethoate quantification in food and soil spiked samples	[207]
T: 2,2-dichlorovinyl dimethyl phosphate M: APTES C: TMOS S: CsPbBr_3_ perovskite P 123	Sol–gel polymerization	2,2-dichlorovinyl dimethyl phosphate	CsPbBr_3_ perovskite coated with mesoporous silica MIP	Fluorescent sensor for 2,2-dichlorovinyl dimethyl phosphate in cabbage and lettuce samples	[208]
T: Chloramphenicol M: APTES C: TEOS S: CDs CTAB	Basic sol–gel polymerization	Chloramphenicol	CDs coated with silica MIP	Dual-emission ratiometric determination of chloramphenicol in milk	[209]
T: Ipradione M: APTMS 1-[3-(Trimethoxysilyl)propyl]urea N-[3-(Trimethoxysilyl)propyl]aniline C: TEOS	Basic sol–gel polymerization	Iprodione	Silica MIP	Silica MIP for iprodione extraction from standard solution	[210]
T: Caffeic acid M: PTEOS/APTMS C: TEOS S: MPTS	Acidic sol–gel polymerization	Caffeic acid	Silica MIP film on Au@MPTS electrode	Electrochemical sensor for determination of caffeic acid in wine samples	[172]
T: Salicylic acid M: APTES/PTMOS C: TEOS	Acidic sol–gel polymerization	Salicylic acid	Silica MIP	Drug delivery carrier	[211]
T-M: TBBPA-ICPTES or BPA-ICPTES C: TEOS S: HMS	Acidic sol–gel polymerization	Bisphenol A	Hollow mesoporous silica MIP	Extraction of trace bisphenol A in real water samples	[212]
T: Cyfluthrin M: APTES/MAA C: TEOS/EGDMA S: Triton X-100/AIBN/FeSe QDs	Reverse microemulsion method	Cyfluthrin	FeSe QDs embedded in silica MIP	Selective and sensitive fluorescent nanosensor for cyfluthrin determination in fish and sediment samples	[213]
T: Norfloxacin M: APTES/MTEOS C: TEOS S: Magnetic halloysite nanotubes	Sol–gel polymerization	Norfloxacin	Magnetic surface imprinted polymer	Extraction of norfloxacin in lake water	[214]
T: Aristolochic acid M: PTMOS C: TEOS S: Magnetic carbon nanotubes	Sol–gel polymerization	Aristolochic acid	Magnetic carbon nanotubes embedded in silicon MIP	Selective removal of aristolochic acid	[215]
T: Gossypol M: APTES C: TEOS S: Silica gel	Acidic sol–gel polymerization	Gossypol	Surface silica MIP on a silica gel support	Selective extraction of Gossypol	[99]
T: Ornidazole M: APTES C: TEOS S: Graphene QDs	Sol–gel polymerization	Ornidazole	Graphene QDs embedded in silica MIP	Luminescence nanosensor for ornidazole detection in biological samples	[216]
T: Gly-Trp M: ABPA-GPTES C: TEOS S: CTAB	Basic emulsion sol–gel polymerization	N-(1-deoxy-D-glucose-1-yl)tryptophan	Molecularly imprinted mesoporous silica nanoparticles	Selective extraction of specific amadori compounds	[217]
T: Gossypol M: APTES C: TEOS S: Silica gel	Sol–gel polymerization	Gossypol	Surface layer imprinted silica MIP	Comparison of organic and inorganic MIP composition	[218]

ABPA-GPTES—aminophenylboronic acid—3-glycidylocy propyl triethoxysilane reaction product; AEAPMS—N-(b-aminoethyl)-g-aminopropyl methyldimethoxy silane; AIBN—azobis(izobutyronitryl); APTES—(3-Aminopropyl)triethoxysilane; APTMS—(3-Aminopropyl)trimethoxysilane; BIMS—product of reaction of 3-mercaptopropionic acid with 3-(trimethoxysilyl) propylmethacrylate; BPA—bisphenol A; BUPTEOS—N-(benzyl)-N′-(3-(triethoxysilyl)propyl)urea; C—cross-linker; CDs—carbon dots; CSPTMS—2-(4-chlorosulfonylphenyl)ethyl trimethoxysilane; CTAB—cetyltrimethylammonium bromide; EGDMA—ethylene glycol dimethacrylate; FITC—fluorescein isothiocyanate; GCE—glassy carbon electrode; Gly-Trp—N-(1-deoxy-D-glucose-1-yl)tryptophan; GO QDs—graphene oxide quantum dots; GYLMO—3-glycidyloxypropyltrimethoxysilane; HMS—hollow mesoporous silica; ICPTES—(3-isocyanatopropyl)triethoxysilane; ITO—indium tin oxide; M—functional monomer; MAA—methacrylic acid; MIP—molecularly imprinted polymer; MOF-177—metal-organic framework 177; MTEOS—methacryloxypropyltrimethoxysilane; MPTS—3-mercaptopropyltimethoxysilane; NP-3-APTMOS—N-phenyl-3-aminopropyltrimethoxysilane; PTEOS—phenyltriethoxysilane; PTMOS—phenyl trimethoxysilane; QDs—quantum dots; S—support; T—template; TBBPA—3,30,5,50-tetrabromobisphenol; TEOS—tetraethoxysilane; TMOS—tetramethoxysilane.
